# Rational Design of WO_3_ Nanostructures as the Anode Materials for Lithium-Ion Batteries with Enhanced Electrochemical Performance

**DOI:** 10.1007/s40820-014-0013-5

**Published:** 2014-11-14

**Authors:** Yang Liu, Yang Jiao, Haiyue Zhou, Xiang Yu, Fengyu Qu, Xiang Wu

**Affiliations:** Key Laboratory for Photonic and Electronic Bandgap Materials, Ministry of Education and College of Chemistry and Chemical Engineering, Harbin Normal University, Harbin, 150025 People’s Republic of China

**Keywords:** WO_3_ nanostructures, Anode materials, Li-ion batteries

## Abstract

A facile, one-step hydrothermal method was employed to synthesize two kinds of WO_3_ nanostructures. By using different kinds of sylvine, tungsten trioxide (WO_3_) with different morphologies of microflowers and nanowires was obtained, respectively. The discharge capacities for microflowers and nanowires are 107 and 146 mAh g^−1^ after 180 cycles, and their corresponding capacity retentions after the first cycle are 72 and 85 %, respectively. Even at a high current density of 1,600 mAh g^−1^, the discharge capacities of WO_3_ microflowers and nanowires are as high as 433 and 557 mAh g^−1^ after 40 cycles, in which the current densities were increased stepwise. It is worth mentioned that the rate capability of the nanowires is superior to that of the microflowers. However, the cycle performance of the microflowers is better than nanowires, revealing that the morphology and structure of the as-synthesized WO_3_ products can exert great influence on the electrochemical performances.

## Introduction

In the past few years, owing to the development of new type energy materials, more and more researchers devote their efforts to investigate high-performance power sources with higher power and energy densities for long time operation, i.e., lithium-ion batteries (LIBs) and supercapacitors (SCs) [1–9]. Especially, the LIBs are obviously superior to the supercapacitors in aspect of energy storage due to the higher energy density [10–12]. Moreover, as one of the most important low-cost, light-weight, highly efficient, and environmentally friendly rechargeable power sources for consumer electronic products, LIBs have attracted worldwide attentions because of the increasing concerns about energy and environmental problems. Therefore, more and more efforts are devoted to develop high performance and miniaturization LIBs [13–15].

It is well known that the electrode materials correlate to the performance of lithium-ion batteries, which is strongly influenced by the sort and the structure of a material [16–18]. Hence, to rational design and synthesize semiconductor nanostructures with desired structures and shapes are a very important task. As an important *n*-type semiconductor, tungsten trioxide (WO_3_) has received a lot of attentions in recent years due to its attractive physiochemical properties and extensive potential applications [19–24].

In this paper, we developed a simple hydrothermal strategy to design and fabricate WO_3_ nanostructures with two different morphologies and investigated their electrochemical performance as anode materials for LIBs. The rate capability of the WO_3_ nanowires was found to be superior to that of the microflowers. However, the cycle performance of the microflowers is better than that of the nanoribbons, revealing the morphology and structure of the as-obtained product might exert great influence on their electrochemical performances.

## Experimental

All chemicals used are analytical grade without further purification. In a typical procedure to prepare WO_3_ microflowers, 12.5 mmol Na_2_WO_4_·2H_2_O was added to 100 mL deionized water. After being stirred for 20 min at room temperature, 3 M HCl was added dropwise to the solution until the pH reached 12, and a yellow transparent solution was formed. Subsequently, 35 mL H_2_C_2_O_4_ was added in the above solution with continuous stirring, and then the solution was diluted to 250 mL.

After that 1.0 g of KCl was added into above 20 mL solution with stirring, followed by transferred into a 40 mL Teflon-lined stainless steel autoclave, and the autoclave was sealed and maintained at 180 °C for 16 h. After the solution was cooled down to room temperature naturally, the as-prepared yellow precipitation was rinsed extensively with deionized water and ethanol, and finally dried in air at room temperature for further characterization. WO_3_ nanowires were synthesized through the same method except 1.0 g of K_2_SO_4_ was added instead of KCl.

## Results and Discussion

**Fig. 1:**
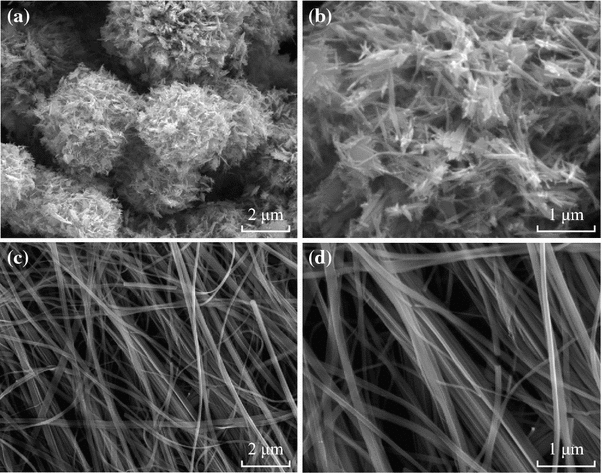
SEM images of the as-synthesized WO_3_ microflowers (**a**–**b**) and nanowires (**c**–**d**) at different magnifications

The morphology and size of the as-product were first characterized by field-emission scanning electron microscope (SEM). A survey view at low magnification (Fig. 1a) reveals that the sample is composed of uniform flower-like microspheres with diameters of ~5 μm. A high-magnification SEM image of a microflower (Fig. 1b) clearly presents that the flower-like nanostructures are assembled from many nanowires with average length of hundreds of nanometers.

When using K_2_SO_4_ to replace KCl with other parameters constant, some nanowires were formed. From Fig. 1c, it can be observed that these nanowires possess a certain orientation, and their average lengths are more than 10 μm. Further observation (Fig. 1d) found that each nanowire has a smooth and uniform surface.

**Fig. 2:**
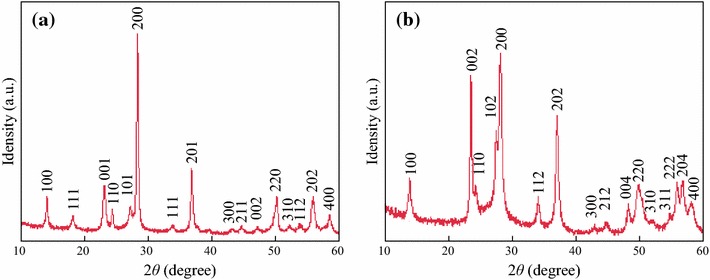
XRD pattern of **a** WO_3_ microflowers and **b** WO_3_ nanowires

The phase and purity of the products were determined by X-ray diffraction (XRD). Figure 2a illustrates the typical diffraction pattern of the microflowers assembled from many nanowires, and all the peaks can be well indexed to the hexagonal WO_3_ structure (JCPDS card NO. 33–1387). No characteristic peaks for other impurities are observed, revealing the high purity of the prepared WO_3_ microflowers. At the same time, each WO_3_ contains 0.33 water of crystallization, which should come from hydrothermal process and could be removed by calcination. Figure 2b shows XRD pattern of the nanowires. All diffraction peaks match well with the standard JCPDS card (no. 85–2460). XRD measurements show that there are no secondary phases or residuals of tungsten. The sharp and strong peaks indicate that WO_3_ nanowires have good crystal quality. The lattice parameters are calculated (*a* = 7.334 Å, *c* = 7.658 Å) and are consistent with the standard values.

**Fig. 3:**
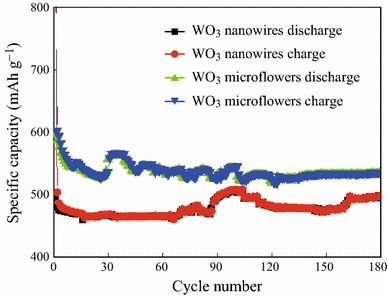
Cycle performances of WO_3_ microflowers and nanowires at 200 mA g^−1^ in the voltage range of 0.01–3 V

To investigate the practical applications of the as-synthesized two WO_3_ nanostructures as the anode materials for the LIBs, a series of electrochemical measurements were conducted. The cycling performance of two structures at a current density of 200 mA g^−1^ with a voltage range of 0.01–3.00 V over 180 cycles is presented in Fig. 3. The first specific discharge capacities of WO_3_ microflowers and nanowires reach 718.8 and 664.3 mAh g^−1^, respectively. It can be identified that both WO_3_ microflowers and nanowires electrodes show a relatively high initial irreversible capacity loss of about 72 and 85 %, respectively, which can probably be attributed to the reduction of WO_3_ to *W* and some side reactions such as formation of the solid-electrolyte interface [25]. It is worth noting that neither microflowers nor nanowires electrodes have large loss of capacity from the second loop. It is apparent that both two samples demonstrate comparably good capacity retention upon extended cycling up to 180 charge/discharge cycles, and the discharge capacity of WO_3_ microflowers electrode is 549.8 mAh g^−1^, while the discharge capacity for the nanowires electrode is about 503.9 mAh g^−1^ after 180 cycles, and the values shown by the as-prepared WO_3_ nanostructure electrodes are higher than that of graphite (372 mAh g^−1^) [26, 27]. This result indicates that WO_3_ nanostructures are promising as anode materials for Li-ion batteries. Besides, the results also demonstrate that the reversible capacity of the microflowers is higher than that of nanowires. Furthermore, the cycle stability of the microflowers is higher than nanowires since 110th cycle, demonstrating that the morphology and the particle size of the as-prepared product have great influence on the electrode performance.

To compare the capacitive performance of the as-synthesized WO_3_ microflowers and nanowires with the previous work [28], a comparsion is made, as shown in Table 1. Obviously, WO_3_ microflowers and nanowires electrodes exhibit the outstanding capacitive properties than that of SnO_2_ nanoparticles.

**Table 1:** A comparison of cycle performances of WO_3_ microflowers, nanowires with SnO_2_ microflowers

Electrode types	WO_3_ microflowers	WO_3_ nanowires	SnO_2_ nanoparticles
Initial discharge capacities	718.8 mAh g^−1^	664.3 mAh g^−1^	664.3 mAh g^−1^
Final discharge capacities	549.8 mAh g^−1^	503.9 mAh g^−1^	664.3 mAh g^−1^
Capacity retention	76.4 %	75.8 %	28.8 %

**Fig. 4:**
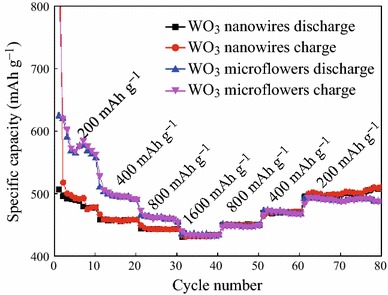
Rate performances at various currents of WO_3_ microflowers and nanowires in the voltage range of 0.01–3 V

The rate performances of the as-obtained WO_3_ microflowers and nanowires are also studied, as shown in Fig. 4. WO_3_ microflowers deliver reversible capacities of 591.6, 510.9, 478.8, and 441.4 mAh g^−1^ at high current densities of 200, 400, 800, and 1,600 mA g^−1^, respectively. In addition, after the current rate returning to 200 mA g^−1^, the electrode delivers a specific discharge capacity of about 502.9 mAh g^−1^. Upon an increase in the discharge rates to 200, 400, 800, and 1,600 mA g^−1^, the reversible capacities of WO_3_ nanowires are maintained at 504.9, 464.6, 450.2, and 435.9 mAh g^−1^, respectively. After the current rate returns to 200 mA g^−1^, the nanowires electrode delivers a specific discharge capacity of ~503.4 mAh g^−1^. From the above results, we can see that when returning to the current density of 200 mAh g^−1^ the capacity of microflowers is less than the initial value, but the capacity of the nanowires almost has no change. This may be attributed to the volume change of the microflowers during the charging and discharging process at large current density. Obviously, WO_3_ nanowires exhibited better rate performance than the microflowers.

Electrochemical measurement results indicated that at least two processes occur for Li^+^ intercalation into WO_3_ nanostructures: a fast Li^+^ insertion between WO_3_ layers and a subsequent slow diffusion and residence of Li^+^ in the interlayer spacing. Li^+^ insertion process occurs in a disorder way and leads to decreased interlayer distance. The slower process causes two dimensional relaxation of the structure within WO_3_ layers. During lithium insertion, tungsten was reduced from high valence to lower oxidation state, resulting in an increase in its coordination number and changes in cell parameters. Significant alteration in crystal structure may be induced if large amount of lithium ions are inserted. The reaction mechanism for WO_3_/Li cell can be described as follows:

1 $${\text{WO}}_{3}+{\;\text{6Li}}^{+}+6e^{-}\to \;W\;+{\;\text{3Li}}_{2}\text{O}$$

2 $$W\;+{\;\text{3Li}}_{2}\text{O}\;\to {\;\text{WO}}_{3}+{\;\text{6Li}}^{+}+6e^{-}$$

Such good rate capability and reversibility of WO_3_ microflowers and nanowires can be attributed to the superior uniform structures and small size of the products that reduce lithium ion diffusion distance and facilitate rapid lithium ion diffusion. Nanoscale particles are able to diffuse much more easily and have better accommodation of structural strain for the electrochemical reaction of lithium, resulting in improving the electrochemical performance. Similar case has also been encountered in the SnO_2_/*α*-MoO_3_ core–shell nanobelts and hierarchical WO_3_ flowers [29, 30]. Moreover, it has been demonstrated that small size effect of WO_3_ nanostructures, arising from an increased total number of surface atoms, can greatly increase the electrochemical reactivity and make the conversion between Li^+^ and Li_2_O reversible [31, 32].

## Conclusion

In conclusion, WO_3_ microflowers and nanowires have been successfully synthesized through a facile hydrothermal process. Potassium salt plays an important role in adjusting the morphology of the products. The as-prepared two WO_3_ structures show significantly improved cycle lives and rate performance due to their unique structures. 
